# Differential attainment at MRCS according to gender, ethnicity, age and socioeconomic factors: a retrospective cohort study

**DOI:** 10.1177/01410768221079018

**Published:** 2022-02-16

**Authors:** Ricky Ellis, Peter A Brennan, Amanda J Lee, Duncan SG Scrimgeour, Jennifer Cleland

**Affiliations:** 1 Institute of Applied Health Sciences, University of Aberdeen, Aberdeen, AB24 3FX, UK; 2 Urology Department, Nottingham University Hospitals, Nottingham, NG5 1PB, UK; 3 Department of Maxillo-Facial Surgery, Queen Alexandra Hospital, Portsmouth, PO6 3LY, UK; 4 Medical Statistics Team, Institute of Applied Health Sciences, University of Aberdeen, AB24 3FX, UK; 5 Department of Colorectal Surgery, Aberdeen Royal Infirmary, Aberdeen, AB25 2ZN, UK; 6 Lee Kong Chian School of Medicine, Nanyang Technological University, 308232, Singapore

**Keywords:** Clinical, medical education, non-clinical, postgraduate, surgery

## Abstract

**Objective:**

A recent independent review on diversity and inclusivity highlighted concerns that barriers to surgical career progression exist for some groups of individuals and not others. Group-level differences in performance at the Intercollegiate Membership of the Royal Colleges of Surgeons (MRCS) examinations have been identified but are yet to be investigated. We aimed to characterise the relationship between sociodemographic differences and performance at MRCS.

**Design:**

Retrospective cohort study.

**Setting:**

Secondary care.

**Participants:**

All UK MRCS candidates attempting Part A (*n = *5780) and Part B (*n = *2600) between 2013 and 2019 with linked sociodemographic data in the UK Medical Education Database (https://www.ukmed.ac.uk).

**Main outcome measures:**

Chi-square tests established univariate associations with MRCS performance. Multiple logistic regression identified independent predictors of success, adjusted for medical school performance.

**Results:**

Statistically significant differences in MRCS pass rates were found according to gender, ethnicity, age, graduate status, educational background and socioeconomic status (all *p* < 0.05). After adjusting for prior academic attainment, being male (odds ratio [OR] 2.34, 95% confidence interval [CI] 1.87–2.92) or a non-graduate (OR 1.98, 95% CI 1.44–2.74) were independent predictors of MRCS Part A success and being a non-graduate (OR 1.77, 95% CI 1.15–2.71) and having attended a fee-paying school (OR 1.51, 95% CI 1.08–2.10) were independent predictors of Part B success. Black and minority ethnic groups were significantly less likely to pass MRCS Part B at their first attempt (OR 0.41, 95% CI 0.18–0.92 for Black candidates and OR 0.49, 95% CI 0.35–0.69 for Asian candidates) compared to White candidates.

**Conclusions:**

There is significant group-level differential attainment at MRCS, likely to represent the accumulation of privilege and disadvantage experienced by individuals throughout their education and training. Those leading surgical education now have a responsibility to identify and address the causes of these attainment differences.

## Introduction

Equity and fairness are fundamental values that must be prioritised within medical training and assessment to create a diverse and inclusive medical workforce.^1^ This is not the case in UK surgery as evidenced by limited diversity within the consultant surgeon population and senior leadership roles, presented in a recent independent review on diversity and inclusivity led by Baroness Helena Kennedy.^1^ This report highlighted widespread concern that barriers to progression in surgical careers exist for some groups of individuals and not others. The first step in addressing the issue – and thus enabling equity and fairness within surgery – is identifying barriers to progression.

Recent research identifying group-level differences, or differential attainment, at the Intercollegiate Membership of the Royal Colleges of Surgeons (MRCS) examinations poses the question of whether this examination is a barrier to career progression for some groups.^2^ Scrimgeour et al.^2^ found that men, White candidates and younger candidates were significantly more likely to pass MRCS at the first attempt, suggesting that individual differences in personal and social circumstances may impact examination outcomes. However, this study included data on a limited number of sociodemographic differences. It was, therefore, unable to adjust for numerous other confounding variables including prior academic performance, which is known to be the best predictor of later success in medical assessments.^3–6^ Therefore, it remains to be seen whether sociodemographic factors such as gender and ethnicity are independently associated with differential attainment at MRCS or whether their association with performance is largely related to other individual differences or ability, as indicated by prior academic attainment.

Successful completion of MRCS Part A (written component) and Part B (objective structured clinical examination) is a prerequisite for entry into UK higher surgical specialty training. The use of MRCS as a ‘gatekeeper’ for entry to higher surgical specialty training means that examination performance is likely to have a significant and lasting impact on the career progression of trainees. These concerns have prompted investigation into differential attainment in postgraduate examinations, including MRCS, to highlight and address potential barriers to career progression for some groups of trainees.^1,7^ In the current study we aimed to characterise and understand the relationship between sociodemographic differences and performance at MRCS. Given that prior academic attainment is known to be the best predictor of later success in medical assessments,^3–6^ we also adjusted analyses for candidates’ performance at medical school to isolate the true relationship between sociodemographic factors and MRCS success.

## Methods

This was a retrospective cohort study using data from the UK Medical Education Database (UKMED) and the four Royal Colleges of Surgeons of the UK and Ireland (Edinburgh, Glasgow, England and Ireland). UKMED links educational outcomes for all trainees within the UK by regularly crosslinking data from a number of sources, including the Higher Education Statistics Authority (HESA) Limited and the General Medical Council. For more information on UKMED please refer to: https://www.ukmed.ac.uk/. Anonymised data were extracted for all UK graduates who attempted either MRCS Part A or Part B between April 2013 and May 2019. The study period was established between these dates to include the maximum number of candidates with educational performance measure (EPM) scores (described below) before the COVID-19 pandemic, thereby eliminating the effect this may have had on training and assessment.

We focused on examining differences between groups on the basis of gender, ethnicity, age and indicators of socioeconomic status.^4,8–16^ The following standardised and anonymised data were extracted from UKMED prior to analysis: gender, ethnicity, age; graduate status at the time of entry to medical school; parental education; parental occupation; participation of local areas (POLAR) quintile (which classifies areas of the UK into categories according to the level of participation of young people in higher education and ranges from quintile 1 (lowest participation in higher education) to 5 (highest participation)); school type; entitlement to income support and free school meals; index of multiple deprivation (IMD) quintile (IMD identifies small zones of deprivation throughout the UK mapped to socioeconomic domains and range from quintile 1 (most deprived) to quintile 5 (least deprived)); and EPM score (see later). First attempt examination scores were used throughout, as these have been shown to be the best predictor of future performance in postgraduate examinations.^2,4,5^

### Measures of socioeconomic status

Variables were linked to MRCS performance on an individual level by UKMED. Measures of educational background included: parental education (university-educated or not); parental occupation (mapped to national statistics socioeconomic codes on a scale of 1 to 5 and dichotomised into managerial and professional occupations (1) versus other occupations (codes 2–5) as used in previous studies).^15,16^ POLAR scores were dichotomised for analysis with POLAR quintiles 1 and 2 representing students from the lowest participation areas versus students from quintiles 3, 4 and 5.^15,16^ High-school education was dichotomised into state (non-fee paying) or fee-paying school.

Measures of socioeconomic status were similar to those used in previous studies^15,16^ and included IMD quintile and entitlement to income support and free school meals. IMD quintiles were dichotomised into 1 and 2 (commonly used in higher education to identify most disadvantaged, or ‘widening participation students’) versus quintiles 3, 4 and 5. The dichotomisation of these variables also results in larger subgroup sizes, maximising statistical power for analyses and enabling more precise estimates of effect sizes. Both POLAR and IMD use UK postcodes for the classification of applicants. Therefore, POLAR and IMD scores were included in analyses only for non-graduate entry medical students, as these were most likely to represent the parental/childhood home (as opposed to a university dwelling for those who had undertaken a prior degree).

Although many non-UK educated doctors sit the MRCS, the focus of our study was on UK graduates because sociodemographic indicators are context-specific. For more information on the measures of socioeconomic status used in this study, please refer to the UKMED data dictionary (https://www.ukmed.ac.uk/).

### Adjustment for prior academic attainment

The performance of medical school graduates in the UK is quantified by an EPM score on completion of medical school. The EPM is the sum of three scores; points awarded for additional degrees (maximum of 5 points), points awarded for publications (maximum of 2 points) and a decile score based on their performance throughout medical school, ranging from 34 points for the 10th (lowest) decile to 43 points for students in the 1st (highest) decile. Previous studies have found that the EPM decile score demonstrates the most predictive value^6,17^ of the three component parts of the EPM. Additionally, points for additional degrees and publications are more accessible to students from more affluent backgrounds, who are able to ‘pay for points’, creating a financial barrier to success.^6^ Therefore, and as per previous studies, EPM decile scores (not EPM total score) were used as a measure of prior academic attainment.^18^

### Statistical analysis

All analyses were conducted using SPSS® v22.0 (IBM, Armonk, NY, USA). Univariate analysis using chi-square testing was initially employed to determine any associations with first attempt MRCS pass/fail outcomes. To avoid a high level of multi-collinearity within regression models, Spearman’s Rho correlation coefficients were first calculated for each measure of socioeconomic status (Supplementary Table 1). Where a high correlation coefficient was found between two variables, only one was entered into logistic regression models as per previous studies (e.g. free school meals was carried forward instead of income support and parental education was carried forward instead of parental occupation).^16^ There was a large statistically significant correlation between age at graduation and being a graduate on entry to medicine (r = 0.50, *p* < 0.001). Of all candidates aged >29 years at graduation from medical school, 80% (390/485) were graduate students, and 98% (4640/4735) of all candidates that did not have a degree prior to starting medicine were aged <29 years at the time of graduating from medical school. Therefore, as these two variables represent the same cohort (i.e. mature candidates vs. their younger peers), graduate status being a binary variable was carried forward in regression models. Missing data are stated where present and analyses were performed on a complete case basis to allow easier interpretation and validation by others.

Initial univariate logistic regression models were developed to identify predictors of success at MRCS at the first attempt. Further regression models were developed to identify predictors of MRCS success that were independent of other sociodemographic factors. The first multivariate model adjusts for sociodemographic factors with little or no missing data. The second also adjusts for a measure of prior academic attainment (EPM decile scores). The third multivariate model adjusts for all sociodemographic factors and the final logistic regression model identifies independent predictors of MRCS success after adjusting for all sociodemographic factors and prior academic attainment. Effect sizes are given as odds ratio (OR) (95% confidence interval [CI]). Potential interactions between significant predictors were also examined and stated where found.

When handling, storing and analysing data, the highest standards of security, governance and confidentiality were maintained. In line with the HESA standards (www.hesa.ac.uk), all counts presented have been rounded to the nearest 5 to ensure person-level anonymity.

## Results

Between April 2013 and May 2019, 5780 candidates attempted MRCS Part A; 48% (2755/5780) passed at their first attempt. A total of 2600 candidates later attempted MRCS Part B with 77% (2000/2600) passing at their first attempt. The sociodemographics of the study cohort are shown in Table 1. Of candidates that attempted MRCS Part A and 90% (2340/2600) Part B 91.6% (5290/5780) and 90% (2340/2600) had matched EPM decile scores, respectively. There was no significant difference in cohort demographics between those with and those without matched EPM decile scores (non-significant *p*-values ranged from 0.053 to 0.854).

**Table 1.** Sociodemographics of study cohort.

	Part A (*n = *5780)			Part B (*n = *2600)		
Variable	Percentage of cohort	Number of candidates	Missing data	Percentage of cohort	Number of candidates	Missing data
Gender						
Male	62.0	3580	0	65.9	1715	0
Female	38.0	2200		34.1	885	
Ethnicity						
White	57.7	3140	340	61.0	1485	165
Mixed	4.3	235		4.3	105	
Asian or Asian British	29.6	1610		26.6	650	
Black or Black British	3.3	180		3.3	80	
Other ethnic groups	5.0	275		4.8	115	
Age at graduation						
<29 years	91.4	5280	0	92.8	2415	0
≥29 years	8.6	500		7.2	185	
Graduate on entry to medicine						
Non-graduate	82.6	4735	50	83.2	2150	15
Graduate	17.4	995		16.8	435	
Parental education						
University-educated parent	70.7	1795	3240	71.7	1080	1095
No university-educated parent	29.3	745		28.3	425	
Parental occupation						
I Managerial and professional occupations	73.6	3015	1680	74.1	1420	685
II–V Other occupations	26.4	1085		25.9	495	
POLAR quintile						
III–V Other neighbourhood	87.4	3845	1380	88.5	1820	545
I–II Low Participation neighbourhood	12.6	555		11.5	235	
School type						
Fee-paying school	33.7	1600	1035	34.1	750	400
State-funded school	66.3	3145		65.9	1450	
Income support						
No	84.2	1985	3420	84.1	1180	1195
Yes	15.8	375		15.9	225	
Free school meals						
No	90.3	2230	3310	91.1	1335	1135
Yes	9.7	240		8.9	130	
IMD quintile						
III–V Least deprived	81.3	3575	1380	84.4	1735	545
I–II Most deprived	18.7	825		15.6	320	

The results of univariate analyses between sociodemographic variables and MRCS first attempt pass rates are shown in Table 2. We found statistically significant associations between MRCS Part A pass rates and gender, ethnicity, age, graduate status, parental education, POLAR quintile, school type, entitlement to free school meals and IMD quintile (all *p* < 0.05). We also found statistically significant associations between MRCS Part B pass rates and ethnicity, age, graduate status, parental occupation, entitlement to income support, free school meals and IMD quintile (all *p* < 0.05). There were no significant differences in MRCS Part A or Part B pass rates between those with and those without matched EPM decile scores (*p* = 0.454 and *p* = 0.557 respectively) (Table 2).

**Table 2.** Univariate analysis of Membership of the Royal College of Surgeons (MRCS) examinations first attempt pass rates by sociodemographic variables.

		Part A (*n = *5780)		Part B (*n = *2600)	
Variable	Mean EPM decile score (SD)	Percentage pass (n)	Percentage fail (n)	Percentage pass (n)	Percentage fail (n)
Gender					
Male	38.2 (2.8)	53.0 (1895)	47.0 (1685)	78.1 (1340)	21.9 (375)
Female	38.4 (2.8)	39.1 (860)	60.9 (1340)	74.7 (660)	25.3 (225)
Missing	*n = *485	*n = *0		*n = *0	
*p*-value	0.024	<0.001		0.054	
Ethnicity					
White	38.7 (2.7)	52.3 (1650)	47.7 (1490)	81.0 (1205)	19.0 (280)
Mixed	38.4 (2.7)	48.7 (115)	51.3 (120)	78.3 (80)	21.7 (25)
Asian or Asian British	37.6 (2.8)	42.3 (680)	57.7 (930)	69.8 (455)	30.2 (195)
Black or Black British	37.4 (2.8)	32.6 (60)	67.4 (120)	69.1 (55)	30.9 (25)
Other ethnic groups	37.6 (2.9)	36.0 (100)	64.0 (175)	70.7 (80)	29.3 (35)
Missing	*n = *445	*n = *340		*n = *165	
*p*-value	<0.001	<0.001		<0.001	
Age at graduation					
<29 years	38.3 (2.8)	49.0 (2585)	51.0 (2695)	77.6 (1875)	22.4 (540)
≥29 years	38.4 (3.0)	34.3 (170)	65.7 (330)	67.9 (125)	32.1 (60)
Missing	*n* = 485	*n* = 0		*n* = 0	
*p*-value	0.010	<0.001		0.002	
Graduate on entry to medicine					
Non-graduate	38.2 (2.8)	49.0 (2320)	51.0 (2415)	77.7 (1675)	22.3 (475)
Graduate	38.7 (2.8)	42.3 (420)	57.7 (575)	72.9 (315)	27.1 (120)
Missing	*n* = 480	*n* = 50		*n* = 15	
*p*-value	<0.001	<0.001		0.028	
Parental education					
University-educated parent	38.1 (2.8)	52.1 (935)	47.9 (860)	75.9 (820)	24.1 (260)
No university-educated parent	37.9 (2.7)	43.3 (325)	56.7 (420)	75.4 (320)	24.6 (105)
Missing	*n* = 325	*n* = 3240		*n* = 1095	
*p*-value	0.368	<0.001		0.856	
Parental occupation					
I Managerial and professional occupations	38.4 (2.8)	51.0 (1535)	49.0 (1480)	80.1 (1140)	19.9 (280)
II–V Other occupations	38.3 (2.8)	47.8 (515)	52.2 (565)	73.8 (365)	26.2 (130)
Missing	*n* = 350	*n* = 1680		*n* = 685	
*p*-value	0.079	0.070		0.004	
POLAR quintile					
III–V Other neighbourhood	38.2 (2.8)	49.4 (1900)	50.6 (1945)	77.6 (1415)	22.4 (405)
I–II Low participation neighbourhood	38.2 (2.8)	43.8 (245)	56.2 (310)	77.1 (180)	22.9 (55)
Missing	*n* = 430	*n* = 1380		*n* = 545	
*p*-value	0.998	0.014		0.857	
School type					
Fee-paying school	38.1 (2.7)	51.7 (830)	48.3 (770)	79.8 (600)	20.2 (150)
State-funded school	38.4 (2.8)	46.9 (1475)	53.1 (1670)	76.6 (1110)	23.4 (340)
Missing	*n* = 425	*n* = 1035		*n* = 400	
*p*-value	0.169	0.002		0.079	
Income support					
No	38.1 (2.7)	50.2 (995)	49.8 (990)	77.4 (915)	22.6 (265)
Yes	37.7 (2.8)	45.6 (170)	54.4 (205)	68.2 (155)	31.8 (70)
Missing	*n* = 315	*n* = 3420		*n* = 1195	
*p*-value	0.062	0.099		0.003	
Free school meals					
No	38.1 (2.7)	50.7 (1130)	49.3 (1100)	77.0 (1025)	23.0 (310)
Yes	37.5 (2.7)	38.5 (90)	61.5 (150)	63.1 (80)	36.9 (50)
Missing	*n* = 325	*n* = 3310		*n* = 1135	
*p*-value	0.030	<0.001		<0.001	
IMD quintile					
III–V least deprived	38.3 (2.8)	50.8 (1820)	49.2 (1755)	78.8 (1370)	21.2 (365)
I–II most deprived	37.7 (2.7)	39.6 (325)	60.4 (500)	70.9 (225)	29.1 (95)
Missing	*n* = 430	*n* = 1380		*n* = 545	
*p*-value	<0.001	<0.001		0.002	
Matched EPM decile score					
Yes	–	47.9 (2530)	52.1 (2760)	77.1 (1805)	22.9 (535)
No	–	46.1 (225)	53.9 (265)	75.5 (195)	24.5 (65)
*p*-value		0.454		0.557	

SD: standard deviation; n: number of candidates.

Initial univariate regression analyses evaluated predictors of success at MRCS at first attempt. Multivariate regression analyses shown in Table 3 found that after adjusting for EPM decile scores as a measure of prior academic attainment, and sociodemographic factors with little to no missing data, being male (OR 2.30, 95% CI 2.01–2.64) or a non-graduate (OR 2.00, 95% CI 1.67–2.38) were the only factors that independently predict MRCS Part A success. Similarly, being male (OR 2.34, 95% CI 1.87–2.92) or a non-graduate (OR 1.98, 95% CI 1.44–2.74) were found to be the only factors that independently predict MRCS Part A success after adjusting for all sociodemographic factors.

**Table 3.** Logistic regression model showing predictors of pass at the first attempt at MRCS Part A for UK medical graduates before and after accounting for prior academic performance.

	Univariate analysis	Multivariate analysis adjusted for gender, ethnicity and graduate status N = 5390	Multivariate analysis adjusted for gender, ethnicity, graduate status and prior attainment N = 4955	Multivariate analysis adjusted for all sociodemographic factors N = 2170	Multivariate analysis adjusted for all sociodemographic factors and prior attainment N = 1880
**Variable**	Odds ratio (95% CI)	Odds ratio (95% CI)	Odds ratio (95% CI)	Odds ratio (95% CI)	Odds ratio (95% CI)
Gender	1.75	**1.79**	**2.30**	**1.81**	**2.34**
Males vs. females	(1.57–1.95)	**(1.60–2.01)**	**(2.01–2.64)**	**(1.54–2.20)**	**(1.87–2.92)**
Ethnicity					
White	Reference	Reference	Reference	Reference	Reference
	Category	Category	Category	Category	Category
Mixed	0.87	0.86	1.02	1.00	1.24
	(0.66–1.13)	(0.66–1.13)	(0.74–1.40)	(0.64–1.57)	(0.73–2.12)
Asian or Asian British	0.67	**0.63**	0.89	**0.59**	0.83
	(0.59–0.75)	**(0.55–0.71)**	(0.76–1.03)	**(0.48–0.73)**	(0.64–1.08)
Black or Black British	0.44	**0.44**	**0.66**	**0.47**	0.68
	(0.32–0.61)	**(0.32–0.60)**	**(0.45–0.98)**	**(0.28–0.81)**	(0.35–1.32)
Other ethnic groups	0.51	**0.47**	**0.65**	0.73	0.94
	(0.40–0.66)	**(0.36–0.61)**	**(0.47–0.88)**	(0.47–1.15)	(0.55–1.60)
Graduate on entry to medicine	1.31	**1.43**	**2.00**	**1.72**	**1.98**
Non-graduate vs. graduate	(1.14–1.50)	**(1.24–1.66)**	**(1.67–2.38)**	**(1.32–2.24)**	**(1.44–2.74)**
Parental Education	1.42	–	–	**1.37**	1.25
University-educated parent vs. no university-educated parent	(1.20–1.69)			**(1.12–1.68)**	(0.98–1.59)
POLAR quintile	1.25	–	–	0.92	1.05
III–V Other neighbourhood vs. I–II Low participation neighbourhood	(1.05–1.50)			(0.69–1.22)	(0.74–1.49)
School type	1.21	–	–	1.09	1.22
Fee-paying school vs. State-funded	(1.07–1.37)			(0.90–1.31)	(0.97–1.54)
Free school meals	1.64	–	–	1.23	1.21
No vs. Yes	(1.25–2.16)			(0.90–1.69)	(0.81–1.81)
IMD quintile	1.58	–	–	1.04	0.88
III–V Least deprived vs. I–II Most deprived	(1.35–1.84)			(0.80–1.34)	(0.64–1.20)
Educational performance measure decile	1.49	–	**1.54**	–	**1.55**
	(1.45–1.53)		**(1.50–1.58)**		**(1.48–1.62)**

Note: First category displayed within each variable was used as the reference.

CI: confidence interval.

Black candidates and candidates from other minority ethnic groups were less likely to pass MRCS Part A at the first attempt when adjusting for gender, ethnicity, graduate status and prior attainment (OR 0.66, 95% CI 0.45–0.98 and OR 0.65, 95% CI 0.47–0.88, respectively). However, after adjusting for all sociodemographic factors including measures of socioeconomic and educational background, these were no longer independently predictive of MRCS Part A outcomes.

Table 4 reveals that after adjusting for prior academic attainment, gender, ethnicity and graduate status, being male (OR 1.27, 95% CI 1.02–1.58) or a non-graduate (OR 1.66, 95% CI 1.26–2.19) were independent predictors of MRCS Part B success. After adjusting for prior academic attainment and all sociodemographic factors including measures of socioeconomic and educational background, being a non-graduate (OR 1.77, 95% CI 1.15–2.71) and having attended a fee-paying school (OR 1.51, 95% CI 1.08–2.10) were found to be independent predictors of Part B success, with gender no longer being a significant predictor (*p* > 0.05). In addition, Asian (OR 0.49, 95% CI 0.35–0.69) and Black candidates (OR 0.41, 95% 0.18–0.92) were significantly less likely to pass MRCS Part B at the first attempt in comparison to their white colleagues.

**Table 4.** Logistic regression model showing predictors of pass at the first attempt at MRCS Part B for UK medical graduates before and after accounting for prior academic performance.

	Univariate analysis	Multivariate analysis adjusted for gender, ethnicity and graduate status N = 2420	Multivariate analysis adjusted for gender, ethnicity, graduate status and prior attainment N = 2185	Multivariate analysis adjusted for all sociodemographic factors N = 1290	Multivariate analysis adjusted for all sociodemographic factors and prior attainment N = 1125
**Variable**	Odds ratio (95% CI)	Odds ratio (95% CI)	Odds ratio (95% CI)	Odds ratio (95% CI)	Odds ratio (95% CI)
Gender	1.21	**1.23**	**1.27**	1.30	1.27
Males vs. females	(0.99– 1.46)	**(1.01–1.50)**	**(1.02–1.58)**	(0.99–1.71)	(0.94–1.72)
Ethnicity					
White	Reference	Reference	Reference	Reference	Reference
	Category	Category	Category	Category	Category
Mixed	0.85	0.84	0.99	0.78	0.95
	(0.52–1.37)	(0.52–1.36)	(0.58–1.68)	(0.39–1.54)	(0.46–1.97)
Asian or Asian British	0.54	**0.51**	**0.58**	**0.41**	**0.49**
	(0.44–0.67)	**(0.41–0.63)**	**(0.46–0.74)**	**(0.30–0.56)**	**(0.35–0.69)**
Black or Black British	0.53	**0.51**	**0.55**	**0.40**	**0.41**
	(0.32–0.86)	**(0.31–0.83)**	**(0.32–0.94)**	**(0.19–0.82)**	**(0.18–0.92)**
Other ethnic groups	0.57	**0.53**	0.65	**0.54**	0.63
	(0.37–0.86)	**(0.35–0.81)**	(0.41–1.02)	**(0.29–0.98)**	(0.33–1.18)
Graduate on entry to medicine	1.30	**1.42**	**1.66**	**1.67**	**1.77**
Non-graduate vs. graduate	(1.03–1.64)	**(1.10–1.82)**	**(1.26–2.19)**	**(1.13–2.46)**	**(1.15–2.71)**
Parental education	1.02	–	–	1.04	1.00
University-educated parent vs. No university-educated parent	(0.79–1.33)			(0.76–1.41)	(0.72–1.41)
POLAR quintile	1.03	–	–	**0.61**	0.63
III–V Other neighbourhood vs. I–II Low participation neighbourhood	(0.75–1.42)			**(0.38–0.97)**	(0.37–1.05)
School type	1.21	–	–	1.33	**1.51**
Fee-paying school vs. State-funded	(0.98–1.51)			(0.98–1.79)	**(1.08–2.10)**
Free school meals	1.96	–	–	1.47	1.40
No vs. yes	(1.34–2.86)			(0.93–2.32)	(0.84–2.33)
IMD quintile	1.52	–	–	1.25	1.24
III–V Least deprived vs. I–II Most deprived	(1.17–1.99)			(0.85–1.83)	(0.81–1.89)
Educational performance measure decile	1.29	–	**1.28**	–	**1.26**
	(1.24–1.34)		**(1.23–1.33)**		**(1.18–1.33)**

Note: First category displayed within each variable was used as the reference.

CI: confidence interval.

Of note, there was no significant change to the final logistic regression model results for MRCS Part B when entitlement to income support and parental occupation were carried forward instead of free school meals and parental education. None of these measures of socioeconomic status were statistically significant independent predictors of MRCS Part B success (*p* > 0.05).

## Discussion

Our findings highlight significant associations between individual differences (sociodemographic factors) and performance at MRCS, despite few of these being independent predictors of MRCS success.

### Gender

Differential attainment describes variation in performance between different groups taking the same assessment. In this study, we found that men were significantly more likely to pass MRCS Part A at the first attempt than women, although gender was not independently associated with MRCS Part B success when all sociodemographic factors were accounted for (see also literature^2,4,6^). Although it is reassuring that no differential attainment was identified in the face-to-face components of the MRCS examination, making examiner discrimination less likely, further work is required in order to rule out gender bias at a question level. Men have also been found to perform significantly better in surgical training appraisals^19^ and Fellowship of the Royal College of Surgeons,^4,20^ indicating that this pattern is widespread. Interestingly, while gender differences in other high-stakes medical assessments are well documented, it is usually women that perform better than men.^8,10,21,22^

Further research is required to ascertain whether men outperform women in surgical assessments or whether structural or systemic factors exist in training and assessment which privilege men. We suspect the latter, given the culture of surgery has long been criticised for its inflexibility, discrimination and lack of female role models.^1,23^ The growing number of female consultant surgeons in many specialties and the increasing election of female surgeons to senior leadership roles may help change culture and patterns of progression in surgery.^1^

### Ethnicity

As per previous studies, MRCS success was associated with ethnicity.^2,6,18^ Our data also reflect patterns in differential attainment seen throughout the wider literature.^24^ White candidates have been found to perform better at school,^3^ medical school^14^ and in almost all postgraduate medical examinations^8^ including the Membership of the Royal College of Physicians,^10,12^ Membership of the Royal College of General Practitioners,^12,13^ Membership of the Royal College of Psychiatrists^11^ and United States Medical Licencing Examination.^9^

The reasons for this attainment gap remain poorly understood. Differential attainment exists despite adjusting for prior academic attainment. This suggests that differential attainment exists at a structural level, with some groups accumulating disadvantage throughout their education,^24,25^ including at the postgraduate level,^26^ which potentially results in the systemic attainment gap seen in postgraduate assessments.

### Maturity

Younger candidates (defined as aged <29 years in previous studies^2,5,27^) and non-graduates performed significantly better at MRCS than their peers. Graduates have been found in other studies to perform at least as well as undergraduate students in medical school examinations^28^ but appear to perform less well throughout postgraduate training^4,8,19,27^ and on other postgraduate examinations.^4,9^ Whether this differential attainment exists as a result of competing time, family and financial demands^27^ or other barriers to success in postgraduate training for older doctors-in-training, or a combination thereof, is currently unknown.

### Educational background

Despite similar prior attainment (EPM decile score), candidates from lower higher-education participation neighbourhoods or who were first-in-family to attend university performed significantly worse at MRCS Part A, while candidates with parents in non-managerial or professional occupations also performed worse at MRCS Part B. This pattern reflects that seen in the wider literature.^15,16,24^ Additionally, candidates who attended fee-paying schools performed significantly better at MRCS Part A and were 51% more likely to pass MRCS Part B on the first attempt. Taking these findings together and reflecting the wider literature,^15,24^ our findings indicate that candidates from more privileged backgrounds do better at MRCS. The reasons for these patterns of performance remain unclear but may be related to differences in opportunities, role modelling, mentoring and educational support.

### Socioeconomic status

Candidates from lower socioeconomic backgrounds also performed less well. Those entitled to free school meals and from the most deprived areas of the UK performed significantly worse at MRCS Part A and Part B, while those eligible for income support also performed significantly worse at MRCS Part B. Candidates from less affluent backgrounds are known to enter university with lower high-school grades and have been found to perform worse at medical school, indicating an accumulation of educational disadvantage over time.^15,16,29^ While it is unlikely (although this remains to be excluded) that MRCS questions are biased against candidates from less affluent backgrounds, surgical training has long been criticised for perpetuating financial barriers to success. Mandatory training courses, conference fees and even the MRCS itself pose significant financial hurdles for trainees. Those from more affluent backgrounds may be able to afford more courses, conferences and other learning opportunities, accruing advantages that may contribute to stronger performance at MRCS.

### Implications

These data reveal differential attainment in MRCS at a group level that cannot be attributed to learner deficit, suggesting that differential attainment may be the result of the assessment itself or variation in learning and training experiences. Further work is required to examine and understand the potentially complex and multifaceted reasons for these differences. Future research should include quantitative work, scrutinising MRCS Parts A and B for signs of unfairness (e.g. question and/or examiner bias) which has been investigated and largely ruled out for other postgraduate examinations in which differential attainment has been found).^12^ In addition, qualitative work is needed to examine differences in the nature of the learning and assessment environment between groups of trainees.

The wider literature suggests that differential attainment at postgraduate assessment is the lens through which we see the accumulation of educational privilege and disadvantage. This privilege and disadvantage may take place at a macro (policy/systemic), meso (institutional/local) and micro (individual) level at each stage of a candidates’ education and training.^30^ This must be borne in mind by those leading surgical education and training who now have a responsibility to act on these data, to identify and address the causes of differential attainment in the MRCS.^1^ It is clear that systemic change is required in surgery if differential attainment and inequity in education and training is to be addressed. There needs to be a move away from the ‘deficit model’ of thinking and acknowledgement that differential attainment seen in the postgraduate setting is likely the result of a combination of inequity in social and educational opportunities and accumulated historical bias and discrimination experienced by some groups of individuals and not others.^1,26^ Key differences in experiences of the medical learning environment by some groups of trainees have been highlighted within the literature and include a lack of belonging, a lack of mentors, coaching and role models in senior leadership positions, reduced social capital and its subsequent limitation on the development of supportive social networks.^1,24,26,30^ This, combined with ongoing reports of bullying, harassment, microaggressions and discrimination, creates a hostile and unsupportive learning environment experienced by some groups of trainees and not others.^1,26,30^ A greater understanding of these issues and how they affect surgical trainees will enable the development of supportive, inclusive and equitable surgical training programmes.

Additionally, these data highlight groups of individuals at increased risk of failing MRCS. Training providers can use these results to provide additional support and resources to those most in need, mitigating some degree of accumulated disadvantage and enabling equitable training and career progression. Such support and resources may reasonably include mentorship programmes, greater flexibility in training programmes and the provision of grants and bursaries to make access to training opportunities and revision resources equitable. While it may be argued that targeted support programmes stigmatise the recipients, the current model of equal support offered at a policy level and remediation training offered only to those who have already failed MRCS at multiple attempts has so far disproportionately benefited some groups and perpetuated differential attainment at the postgraduate level. There is a paucity of studies looking at the effectiveness of actions designed to address postgraduate differential attainment; therefore, it is imperative that any future interventions are sustainable, have clearly defined outcome measures that aim to reduce differential attainment and are audited regularly for their effectiveness.

### Strengths and limitations

To our knowledge, this large cohort study is the first to assess the relationship between specific sociodemographic factors and MRCS success after adjusting for measures of prior academic attainment. Our findings provide a starting point for understanding how the accumulation of social and educational disadvantage or privilege can impact MRCS performance.

Cohort studies using large datasets are inevitably limited by the data that are available and the limits of statistical analysis. For example, ethnicity was categorised within the dataset into one of five groups to maximise the sample size and resultant statistical power for comparisons. While pragmatic, this approach has been criticised for failing to recognise the diversity and intersectionality of identities and experiences within such broad groupings.^24^ Similarly, the term ‘Black and Minority Ethnic groups’ is not granular but mirrored terminology used in previous research and has allowed us to contextualise and compare the study findings. Despite the UKMED database containing data from a number of sources, a degree of missing data for sociodemographic variables is inevitable and it can be argued that this may limit the generalisability of the results. However, the UKMED database is one of the most comprehensive sources for longitudinal medical education data, providing a unique opportunity to conduct analyses on a complete case basis with no multiple imputation performed, allowing comparability with future studies. Sensitivity analyses were performed including multivariate regression analyses adjusting for sociodemographic factors with little or no missing data. Comparison of these with full multivariate regression analyses that included all factors revealed similar findings between these groups ruling out bias caused by missing data in the subgroup analyses.

As in previous studies, the outcome measure of pass/fail was used since this is what is meaningful to those sitting MRCS.^2,6,18^ Individual MRCS question and station data were not available, potentially hiding group-level variation in performance. Future studies aiming to rule out bias in questioning will require this data to enable a more forensic analysis using differential item functioning.

A-level (high-school exit examination) scores were considered an alternative measure of prior academic attainment. However, continued grade inflation limits the spread and predictive value of A-levels (resulting in the increased reliance on admissions testing for medical school selection purposes) and A-level performance is known to be influenced by external factors such as social class and educational background. Given this, we believe EPM decile scores were the most suitable measure of prior academic attainment in this study.

Finally, our focus was UK graduates because of the availability of socioeconomic measures for this group. International medical graduates are known to exhibit significant differences in performance in many postgraduate examinations compared to UK graduates. Further work is required to investigate whether differential attainment exists for international graduates at MRCS. This will likely require a significantly larger study population to enable meaningful statistical analyses given the likelihood of higher levels of missing sociodemographic and prior academic attainment data, and the difficulty of comparing across contexts.

## Conclusions

This study identified significant differences in MRCS performance between sociodemographic groups, likely to represent the accumulation of privilege and disadvantage experienced by individuals throughout their education and training. Further work is required to identify the causes of this differential attainment, rule out bias at MRCS and examine differences in the nature of the learning environment between groups of trainees.

## Supplemental Material

sj-pdf-1-jrs-10.1177_01410768221079018 - Supplemental material for Differential attainment at MRCS according to gender, ethnicity, age and socioeconomic factors: a retrospective cohort study Click here for additional data file. Supplemental material, sj-pdf-1-jrs-10.1177_01410768221079018 for Differential attainment at MRCS according to gender, ethnicity, age and socioeconomic factors: a retrospective cohort study by Ricky Ellis, Peter A Brennan, Amanda J Lee, Duncan SG Scrimgeour and Jennifer Cleland in Journal of the Royal Society of Medicine

## Declarations

### Guarantor

RE.
