# Supplementary material for: Differential attainment at MRCS according to gender, ethnicity, age and socioeconomic factors: a retrospective cohort study
Source: J R Soc Med. 2022 Feb 16;115(7):257–72. doi: 10.1177/01410768221079018 (PMC9234885; doi:10.1177/01410768221079018)

Supplementary Table 1. Spearman's Rho correlation coefficient matrix including all markers of socioeconomic status. All correlation coefficients demonstrated statistical significance P<0.001.

	School Type	Income Support	Free School Meals	Parental Degree	Parental Occupation	IMD Quintile	Polar Quintile
School Type	1.00	-0.16	-0.12	-0.16	-0.14	-0.12	-0.08
Income Support	-0.16	1.00	0.57	0.25	0.26	0.26	0.19
Free School Meals	-0.12	0.57	1.00	0.20	0.17	0.26	0.17
Parental Degree	-0.16	0.25	0.20	1.00	0.38	0.15	0.18
Parental Occupation	-0.14	0.26	0.17	0.38	1.00	0.17	0.13
IMD Quintile	-0.12	0.26	0.26	0.15	0.17	1.00	0.36
Polar Quintile	-0.08	0.19	0.17	0.18	0.13	0.36	1.00

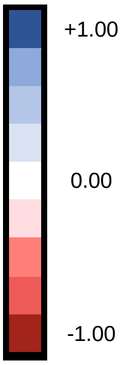

Supplement: sj-pdf-1-jrs-10.1177_01410768221079018 - Supplemental material for Differential attainment at MRCS according to gender, ethnicity, age and socioeconomic factors: a retrospective cohort study [file sj-pdf-1-jrs-10.1177_01410768221079018.pdf]
